# Characterization of advanced glycation end products and their receptor (RAGE) in an animal model of myocardial infarction

**DOI:** 10.1371/journal.pone.0209964

**Published:** 2019-01-11

**Authors:** Bianca de Moraes Fracasso, Juliana Oliveira Rangel, Alessandra Gonçalves Machado, Fernanda Severo Curuja, Amanda Lopes, Virgílio Olsen, Nadine Clausell, Andreia Biolo, Luis Eduardo Rohde, Michael Andrades

**Affiliations:** 1 Cardiovascular Research Laboratory, Experimental Research Center, Hospital de Clínicas de Porto Alegre, Porto Alegre, Rio Grande do Sul, Brazil; 2 Post-Graduate Program in Cardiology and Cardiovascular Science, Universidade Federal do Rio Grande do Sul, Porto Alegre, Rio Grande do Sul, Brazil; Scuola Superiore Sant’Anna, ITALY

## Abstract

Circulating advanced glycation end products (AGE) and their receptor, RAGE, are increased after a myocardial infarction (MI) episode and seem to be associated with worse prognosis in patients. Despite the increasing importance of these molecules in the course of cardiac diseases, they have never been characterized in an animal model of MI. Thus, the aim of this study was to characterize AGE formation and RAGE expression in plasma and cardiac tissue during cardiac remodeling after MI in rats. Adult male Wistar rats were randomized to receive sham surgery (n = 15) or MI induction (n = 14) by left anterior descending coronary artery ligation. The MI group was stratified into two subgroups based on postoperative left ventricular ejection fraction: low (MI^lowEF^) and intermediate (MI^intermEF^). Echocardiography findings and plasma levels of AGEs, protein carbonyl, and free amines were assessed at baseline and 2, 30, and 120 days postoperatively. At the end of follow-up, the heart was harvested for AGE and RAGE evaluation. No differences were observed in AGE formation in plasma, except for a decrease in absorbance in MI^lowEF^ at the end of follow-up. A decrease in yellowish-brown AGEs in heart homogenate was found, which was confirmed by immunodetection of N-ε-carboxymethyl-lysine. No differences could be seen in plasma RAGE levels among the groups, despite an increase in MI groups over the time. However, MI animals presented an increase of 50% in heart RAGE at the end of the follow-up. Despite the inflammatory and oxidative profile of experimental MI in rats, there was no increase in plasma AGE or RAGE levels. However, AGE levels in cardiac tissue declined. Thus, we suggest that the rat MI model should be employed with caution when studying the AGE-RAGE signaling axis or anti-AGE drugs for not reflecting previous clinical findings.

## Introduction

Acute myocardial infarction (AMI) often occurs by atherosclerotic-mediated obstruction of the coronary arteries, which reduces or altogether prevents nutrient transport to cardiomyocytes, leading to cell death and impaired cardiac function. The myocardial ischemia triggers inflammatory signaling, which is followed by attraction of immune cells, production of reactive oxygen species, and molecular damage [1].

Advanced glycation end products (AGEs) are naturally occurring byproducts of human metabolism and can be increased by exposure to exogenous (diet and pollution) [2,3] or endogenous sources (inflammation or hyperglycemia) [4,5]. The process starts with a non-enzymatic reaction between a reducing sugar (e.g. glucose) and an amino moiety from a protein. Further chemical rearrangements will yield heterogeneous compounds with either a yellowish-brown color, fluorescence or both characteristics [6], which can be measured in vitro and in vivo. AGEs are harmful because they cause protein malfunctioning and trigger pro-inflammatory cascades, by activating the AGE receptor (RAGE) [7,8].

Clinical trials and observational studies have shown that increased AGE and RAGE levels in patients with cardiovascular disease are associated with worse prognosis [9,10], and that post-AMI patients with increased plasma fluorescent AGE levels are at higher risk of developing heart failure (HF) [11]. Independent of AGE levels in plasma, soluble RAGE (sRAGE) is positively associated with NYHA class and plasma levels of NT-proBNP, a known marker of HF severity [12]. Despite the increasing importance of glycated molecules in the course of cardiac diseases, the generation of AGEs and expression of their receptor have not been characterized in a representative animal model of permanent myocardial ischemia. Experimental models provide important information on morphology, biochemistry, and electrophysiology in many areas. Our hypothesis is that AGEs and RAGE increase in an experimental model of myocardial infarction (MI) in rats. Thus, the aim of the present study was designed to characterize AGE formation and RAGE expression in plasma and cardiac tissue throughout the cardiac remodeling process in rats subjected to MI.

## Materials and methods

### Reporting guidelines

This manuscript was prepared according to ARRIVE guidelines for reporting animal research [13].

### Animal model and experimental design

One hundred and five male Wistar rats (13–15 weeks old) were randomly allocated into two groups, sham surgery or MI groups, using WinPepi software. After applying exclusion criteria (MI < 30%, sham groups with signal of akinesia/hypokinesia) and discounting deaths (46 deaths occurred between the anesthesia and animal recovery), 29 animals were included in the study (Sham, n = 15; MI, n = 14) (S1 Fig). All animals were weighed one day prior to surgery and at 2, 30, and 120 days postoperatively. Before surgery, the rats were anesthetized with ketamine (100 mg/kg i.p.) and xylazine (10 mg/kg i.p.) and mechanically ventilated (66 BPM and volume of 2.46 mL) (Harvard Model 683). Bupivacaine was injected (1 mg/kg i.m.) over the nerve branch supplying the ribs (two ribs caudal and two ribs rostral to the incision site), and a left lateral thoracotomy was performed between the third and fourth intercostal spaces. The left atrium was pushed aside and the left coronary artery was ligated with 6–0 mononylon thread between the takeoff of the pulmonary artery and the left atrium. The muscle layers and skin incision were sutured with 5–0 mononylon [14]. In sham-operated animals, the procedure was identical, except that arterial ligation was omitted. All animals received tramadol (5 mg/kg i.p.) every 12 hours for a total of three doses, for postoperative analgesia. The rats were housed at constant room temperature (22 ± 2°C) and controlled humidity (40–60%), under a 12:12 h light-dark cycle, with free access to standard chow and water, for 120 days. This study protocol was approved by the institutional ethics committee (Comissão de Ética no Uso de Animais em Pesquisa do Hospital de Clínicas de Porto Alegre, #13–0445).

### Plasma collection and processing

Blood was collected from all animals (2 mL via retro-orbital plexus puncture into tubes containing 50 U heparin) 7 days before surgery and at 2, 30, and 120 days postoperatively. Heparinized blood was centrifuged (3,000 × *g* for 12 min) and plasma was stored at −80°C.

### Tissue collection and processing

After 120 days, the rats were killed by isoflurane overdose and the heart, liver, and lung were harvested. The organs were quickly washed in iced 0.9% NaCl solution and were gently blotted against a clean paper. The heart was weighed and divided visually into three areas: infarcted, peri-infarcted (a 2 mm interface between infarcted and remote areas), and remote (healthy tissue). All were immediately frozen in liquid N_2_ and stored at −80°C. The liver and lung were weighed and placed in an oven at 60°C. After 10 days, they were weighed again for evaluation of congestion. Homogenates were prepared in two different ways according to subsequent technique. For ELISA assays, samples (100 mg) were homogenized in 1 mL of phosphate-buffered saline with 0.05% Tween 20 (pH 7.4) at 4°C, supplemented with protease inhibitor (SIGMAFAST Protease Inhibitor, #S8820, Sigma-Aldrich). For spectrophotometric and dot blot assays, samples (100 mg) were homogenized in 500 μL of phosphate-buffered saline (pH 7.4) at 4°C. Homogenization was performed mechanically, and homogenates were centrifuged (2,400 × *g* for 20 min at 4°C) and stored at −80°C.

### Echocardiography

Left ventricular (LV) function and infarct size were evaluated by echocardiography (HD7 Philips Systems) with a 12–3 MHz linear transducer at 2, 30, and 120 days after surgery. Briefly, after induction of anesthesia with isoflurane (3%, 0.5 L of O_2_/min), the animals were placed on a warming blanket and their chests were shaved. Echocardiography was performed in blinded fashion by an investigator not involved in the surgical procedure.

Infarct size (%MI) was assessed by measuring the akinetic and/or hypokinetic portion (AHP) length of the ventricular walls and expressed as a percentage of the total endocardial perimeter (EP) in three transverse sections of the LV (edges of the mitral valve, papillary muscles, and apical region), using the formula: %MI = (AHP/EP) x 100. Ejection fraction (EF) was calculated by the formula: EF = (LV end-diastolic diameter³ − LV end-systolic diameter³/LV end-diastolic diameter³) x 100, determined in M-mode [15]. Fraction shortening (FS) was estimated by M-mode diameters.

To improve sample homogeneity, animals with a small MI area (< 30%) at day 2 were excluded from the study.

### Reactive free amine content

Protein glycation occurs through reaction of a reducing sugar with a protein amine moiety. Measurement of protein free amines is a proxy for this type of modification. For this purpose, plasma and homogenates were diluted 200- and 100-fold, respectively, with 50 mM carbonate buffer (pH 10.5). Then, 18-μL aliquots of the diluted specimens were pipetted into a 96-well microplate and 182 μL of *o*-phthalaldehyde (OPA) reagent was added in all wells. OPA reagent contains 5 mg OPA (#P1378, Sigma-Aldrich), 100 μL pure ethanol, 5 μL β-2-mercaptoethanol, and 10 mL 50 mM carbonate buffer (pH 10.5). OPA was always used fresh (within 2 h of preparation) and kept from light. Samples were read at 340 nm excitation and 455 nm emission wavelengths within 1.5 min of reaction in a SpectraMax M3 Multi-mode Microplate Reader (Molecular Devices).

### Protein carbonyl content

Protein carbonylation is an index of oxidation of amino groups in proteins, usually caused by oxidative stress or reaction with reducing sugars (glycations/glycoxidation). Protein carbonyl content was determined through reaction of the specimen proteins with 2,4-dinitrophenylhydrazine (DNPH) in 2 M HCl to form chromophoric dinitrophenylhydrazones. Briefly, a sample volume containing 0.6 mg of proteins was reacted with DNPH for 30 min. Proteins were then pelleted with cold 10% trichloroacetic acid (final concentration), washed three times with ethanol: ethyl acetate, and centrifuged (11,000 × *g*, 3 min, 4°C), and dissolved in 1 mL of 6 M aminoguanidine hydrochloride. Absorbance values were recorded at 380 nm, and carbonyl concentration was determined using the DNPH molar extinction coefficient (ε = 22,000 M^−1^ cm^−1^) [16].

### AGE fluorescence and absorbance

Glucose, fructose, ribose, methylglyoxal, glycolaldehyde-derived AGEs may act as fluorophores/chromophores. To assess this characteristic, plasma was diluted 500-fold with phosphate-buffered saline, as previously described [17]. Homogenates were diluted 50-fold with distilled water. The wavelengths were set at 340 nm for AGE absorption [18] and 370 nm/445 nm (excitation/emission) for fluorescence [17]. Both were recorded in a SpectraMax M3 Multi-mode Microplate Reader (Molecular Devices).

### Dot blot analysis

For dot blot analysis, 2G11 antibody (anti-N-ε-carboxymethyl-lysine [CML]) [19] was employed. The antibody was kindly donated by Dr. Ryoji Nagai, from Tokai University, Japan.

Two hundred microliters of diluted plasma (3 μg protein) and homogenate (1 μg protein) were adsorbed in a nitrocellulose membrane using a Bio-dot apparatus (Bio-Rad). The membranes were not blocked, but allowed to dry with methanol before incubation with 2G11 antibody at room temperature, diluted 1:5,000 in Tris-buffered saline supplemented with 0.1% Tween (TTBS) and 1% BSA for 10 min in a SNAP i.d. 2.0 apparatus (Millipore Corporation). Then, membranes were washed four times with TTBS and incubated with secondary anti-mouse antibody (#A4416, Sigma-Aldrich) at 1:15,000 dilution for 5 min at room temperature in SNAP. Color was developed on the spots using the Immobilon Western Chemiluminescent HRP Substrate (Millipore Corporation). Bands were visualized in ImageQuant LAS500 (GE Heathcare Life Sciences) and quantified by optical densitometry using ImageJ analysis software.

### Western blot analysis

Plasma was diluted in 0.1 M Tris (pH 6.8) and Laemmli buffer (20% β-mercaptoethanol) to a final concentration of 3 μg/μL. A 10-μL aliquot of sample was applied to a polyacrylamide gel (SDS-PAGE) and transferred to a PVDF membrane (#IPVH000010, Merk-Millipore). Coomassie Blue staining was performed and recorded in an imager (LAS500, Heathcare GE Life Sciences). After washing with TTBS, membranes were blocked for 1 hour at room temperature and incubated with specific primary antibody (anti-RAGE, #37647, Abcam) at 1:3,000 dilution in TTBS and in 5% BSA, under constant agitation, overnight at 4°C. Then, membranes were washed 4 times with TTBS and incubated with secondary anti-rabbit antibody at 1:10,000 dilution (#7074S, Cell Signalling Biotechnology) for 2 h at room temperature, and washed again after incubation. Color was developed on the bands using the Immobilon Western Chemiluminescent HRP Substrate (Millipore Corporation). Fluorescence was recorded in an ImageQuant LAS500 imager (Heathcare GE Life Sciences) and quantified by optical densitometry using ImageJ analysis software.

### ELISA

RAGE levels were measured using a commercial kit (Rat RAGE/AGER ELISA, #RAB0009, Sigma-Aldrich). Briefly, plasma was used in concentrated form and the assay was performed as described in the manufacturer’s protocol.

### Estimation of total protein content

The protein content of samples was determined by the Bradford method (Bio-Rad Protein Assay, #500–0006, Bio-Rad) using bovine serum albumin (#A9647, Sigma-Aldrich) as the standard [20].

### Data analysis and statistics

Statistical analyses were conducted in IBM SPSS Statistics for Windows, Version 21.0 (IBM Corp). The MI group was stratified into two subgroups based on the median ejection fraction estimated 2 days after surgery (EF_median_ = 53.7%), as follows: MI^intermEF^ (left ventricular ejection fraction [LVEF] above the median; EF mean = 57.7, n = 7); and MI^lowEF^ (LVEF below the median; LVEF mean = 46.2, n = 7). Variables were tested for normality using the Shapiro-Wilk test. To compare groups at the end of follow-up, ANOVA followed by Tukey’s post hoc test (for parametric data) or the Kruskal-Wallis test followed by Dunn’s procedure (for nonparametric data) were used. Non-homogenous or non-normally distributed variables were log-transformed and the geometric mean ± 95% confidence interval (CI) used for analysis. To compare groups over time and within-group variation during follow-up, generalized estimating equations (GEE) were used. Spearman’s correlation (ρ) test was employed to check the association between two variables. Differences were considered significant if p < 0.05.

## Results

### Body weight, cardiac hypertrophy, and cardiac function

**Fig 1:**
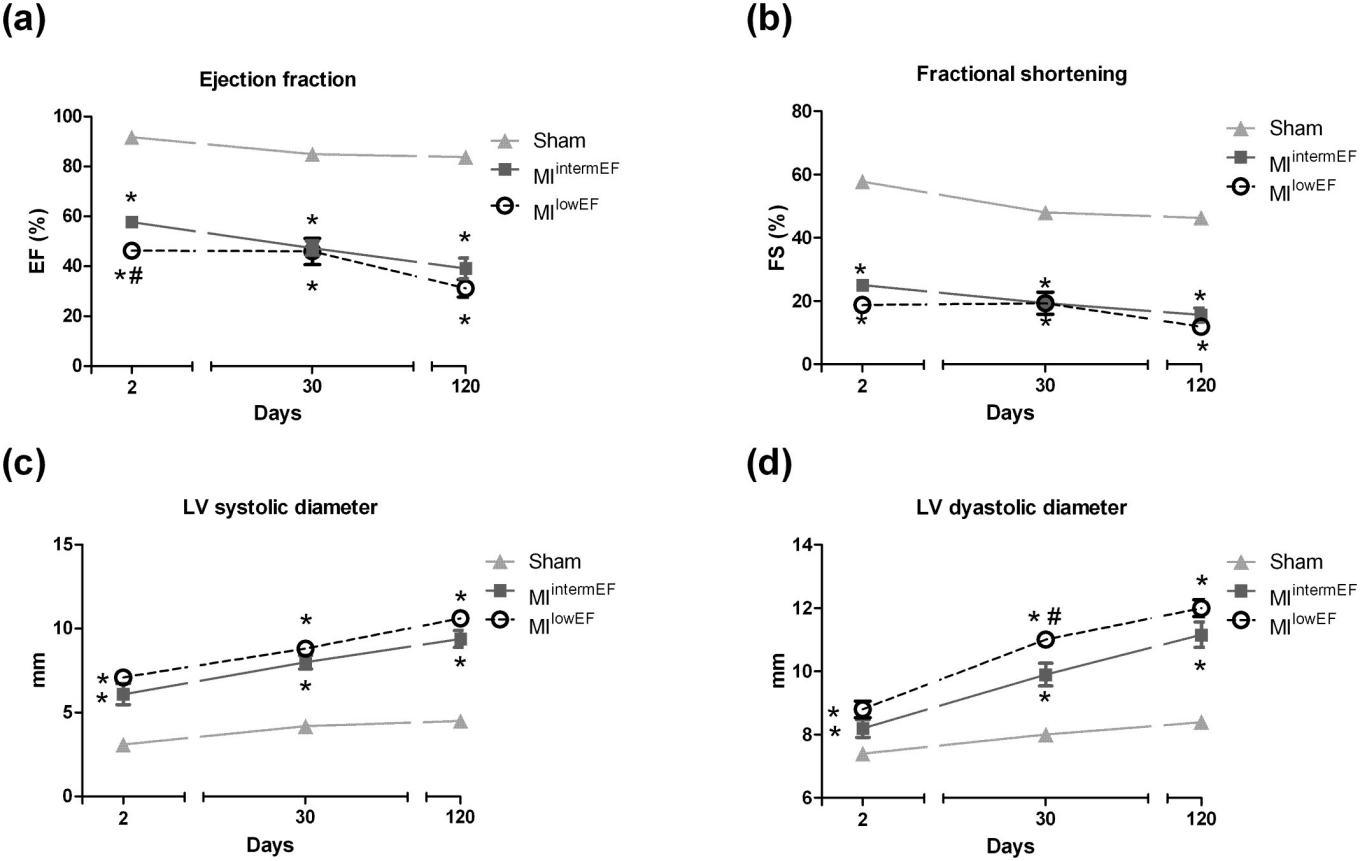
Echocardiographic data collected over the time. Ejection fraction (a), Fractional shortening (b), LV systolic volume (c), and LV diastolic volume (d) were recorded up to 120 days of follow-up. Generalized estimating equations. Data expressed as mean ± SE. * Difference vs. sham; # difference vs. MI^intermEF^ (p < 0.01; sham group, n = 15; MI group, n = 7 per group). MI^intermEF^: EF > 53.7% and < 66.5%; MI^lowEF^: EF < 53.7.

There was no difference in body weight before and after 120 days of surgery (Table 1). AMI surgery yielded hearts markedly damaged (44% and 47% akinetic/hypokinetic in the MI^intermEF^ and MI^lowEF^, respectively) at the end of the follow up. Heart hypertrophy was also evident in rats with a 49% increase in heart weight for MI^lowEF^ animals and a 20% for MI^intermEF^ animals. As expected, LVEF and FS were worse in the MI groups than in the sham group, demonstrating systolic dysfunction (Fig 1a and 1b). In the same way, we saw a progressive increase in LV diameters over the time (Fig 1c and 1d). Despite the difference in LVEF and FS at the start of the protocol, no difference could be detected 120 days later when comparing MI groups with different EF.

**Table 1:** Body weight, organ weight, infarct size, and wall thickness during the follow-up.

	Sham (n = 15)	MI^intermEF^ (n = 7)	MI^lowEF^ (n = 7)
Body weight—initial (g) ^a^	407 ± 8	362 ± 29	397 ± 12
Body weight—final (g) ^a^	524 ± 11	493 ± 21	501 ± 18
Infarct size—initial (%) ^a^	—	44 ± 1.1 **	45 ± 1.8 **
Infarct size—final (%) ^a^	—	44 ± 2.3 **	47 ± 2.2 **
PWTs—initial (mm) ^b^	3.1; 0.67	2.7; 0.38 *	2.8; 1.0 *
PWTs—final (mm) ^b^	2.9; 0.66	2.3; 0.90 ^#^	1.9; 0.71 ^#^
Heart weight/final weight (mg/g) ^b^	2.2; 0.28	2.7; 0.85 **	3.3; 0.80 **

PWTs—posterior wall thickness in systole.

^a^ ANOVA followed by Tukey’s test (mean ± SE).

^b^ Kruskal-Wallis (median; IQR).

^#^p = 0.069;

*p<0.05;

**p < 0.001 vs. sham. MI^intermEF^: EF > 53.7% and < 66.5%; MI^lowEF^: EF < 53.7.

### Measurement of compounds related to AGE formation

**Fig 2:**
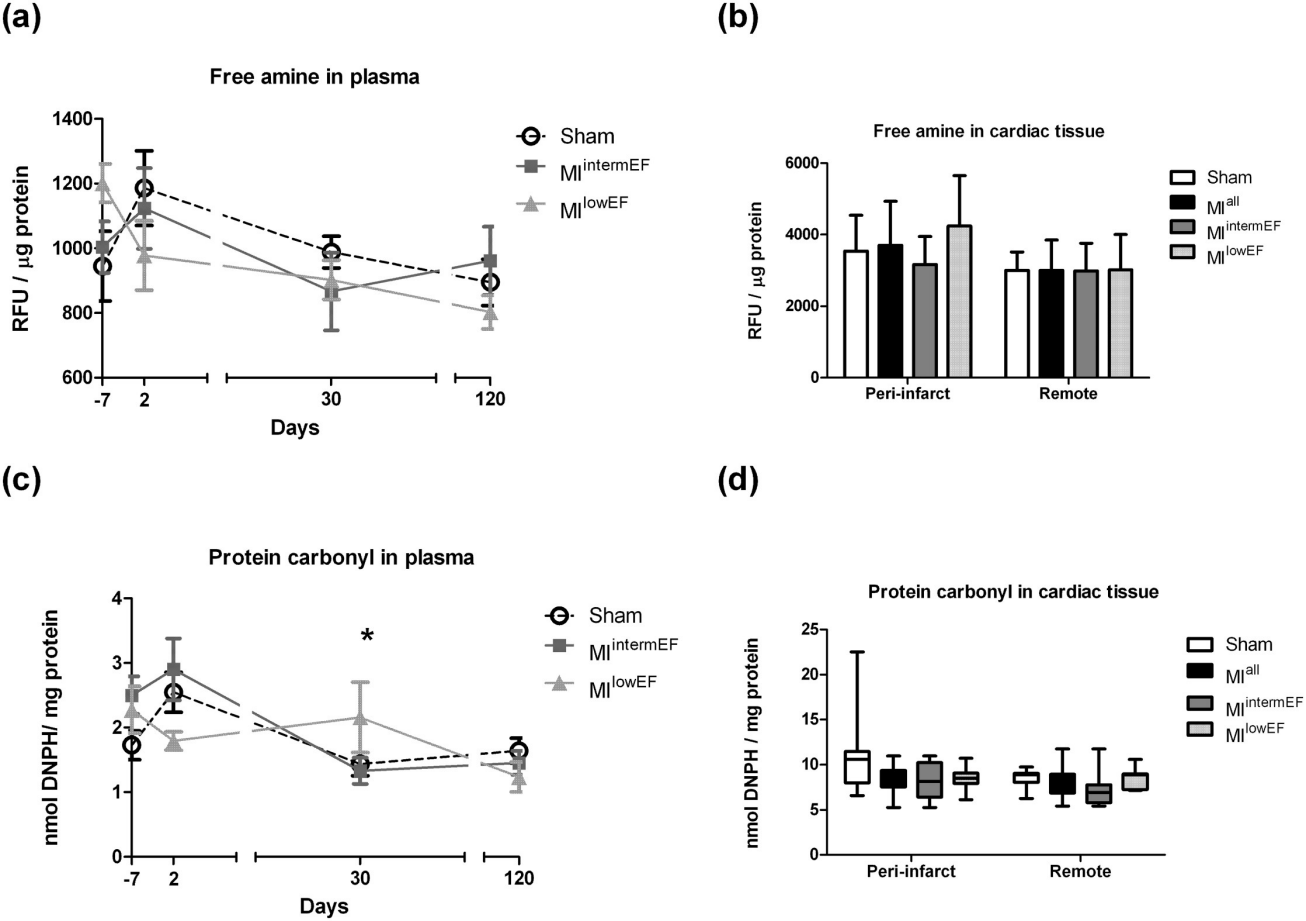
Reactive free amine and protein carbonyl levels. Reactive free amine levels in plasma (a) and in cardiac tissue (b). Protein carbonyl in plasma (c) and in cardiac tissue (d). Statistical analysis: GEE (a and c), data expressed as mean ± SE; ANOVA with Tukey’s test (b), data expressed as mean ± standard deviation (SD); Kruskal-Wallis with Dunn test (d), data expressed as median ± SE. * Difference vs. MI^intermEF^ (p < 0.05; sham group, n = 15; MI group, n = 7 per group). RFU: relative fluorescence units; DNPH: 2,4-dinitrophenylhydrazine. MI^intermEF^: EF > 53.7% and < 66.5%; MI^lowEF^: EF < 53.7.

As shown in Fig 2, reactive free amine content in plasma (Fig 2a) and in peri-infarct and remote myocardial areas (Fig 2b) was similar between groups. Cardiac tissue homogenates were also compared for protein carbonyl content, and no significant difference was found (Fig 2d). Protein carbonyl plasma levels were increased at 30 days in MI^lowEF^ compared with MI^intermEF^ animals. However, no difference was detected at the end of follow-up (Fig 2c).

### Yellowish-brown AGE content decreases in peri-infarct myocardium after MI

**Fig 3:**
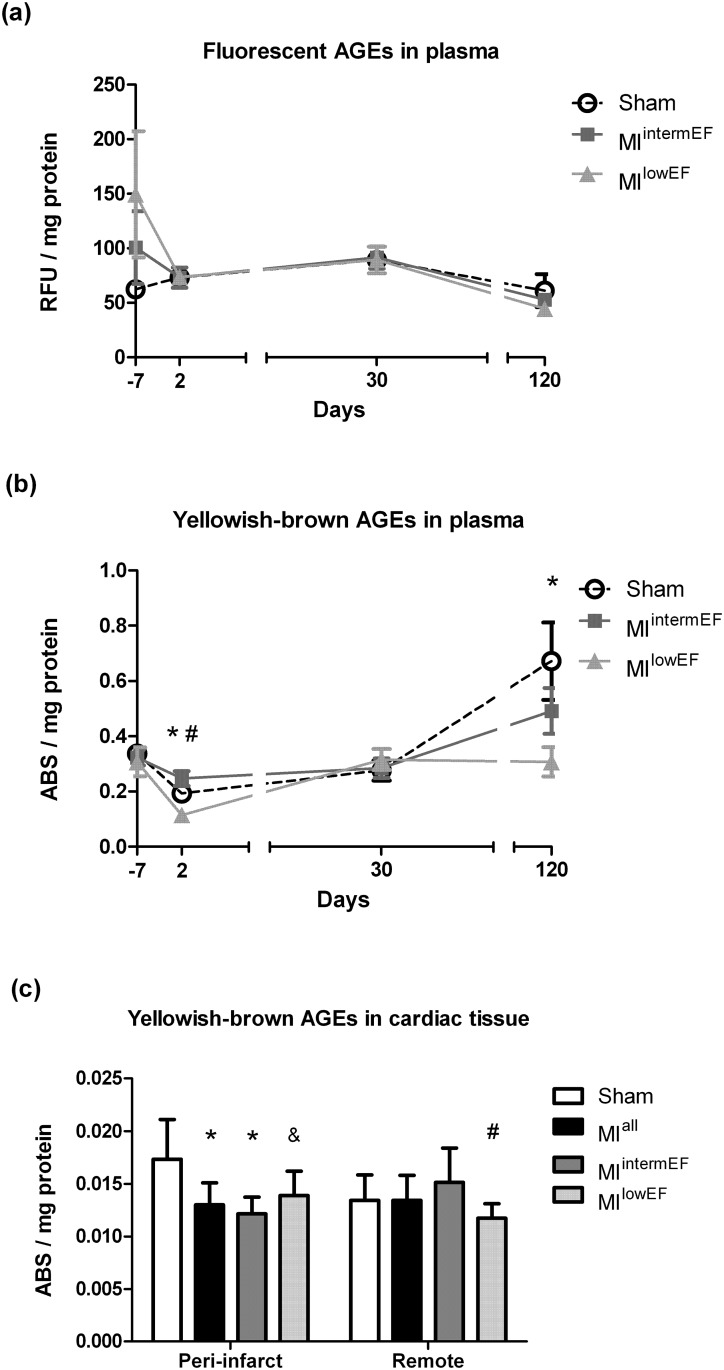
Fluorescent and yellowish-brown AGE levels. Fluorescence in plasma (a). Absorbance in plasma (b) and in cardiac tissue, both peri-infarct and remote (c). Statistical analysis: GEE (a and b), data expressed as mean ± SE; ANOVA with Tukey’s test (c), data expressed as mean ± SD. *Difference vs. sham; # difference vs. MI^intermEF^ (p < 0.05; sham group, n = 15; MI group, n = 7 per group). & Difference vs. sham (p = 0.053). ABS: absorbance; RFU: relative fluorescence units. MI^intermEF^: EF > 53.7% and < 66.5%; MI^lowEF^: EF < 53.7.

Plasma fluorescent AGE levels during follow-up did not differ among groups (Fig 3a). Unexpectedly, plasma levels of yellowish-brown AGEs decreased in MI^lowEF^ animals at 2 and 120 days after surgery when compared with the sham group (Fig 3b). In peri-infarct heart tissue, yellowish-brown AGE levels decreased by 23% in MI group (p = 0.001). This decrease was more evident in the MI^intermEF^ group (-29%; p = 0.003) than in the MI^lowEF^ group (-18%; p = 0.053) compared with the sham group. In remote myocardial tissue, yellowish-brown AGE levels decreased in MI^lowEF^ compared to MI^intermEF^ animals (Fig 3c). Fluorescence detection in tissue homogenates could not be performed due to lack of sensitivity of the assay for this type of sample.

### CML in plasma and tissue homogenates

**Fig 4:**
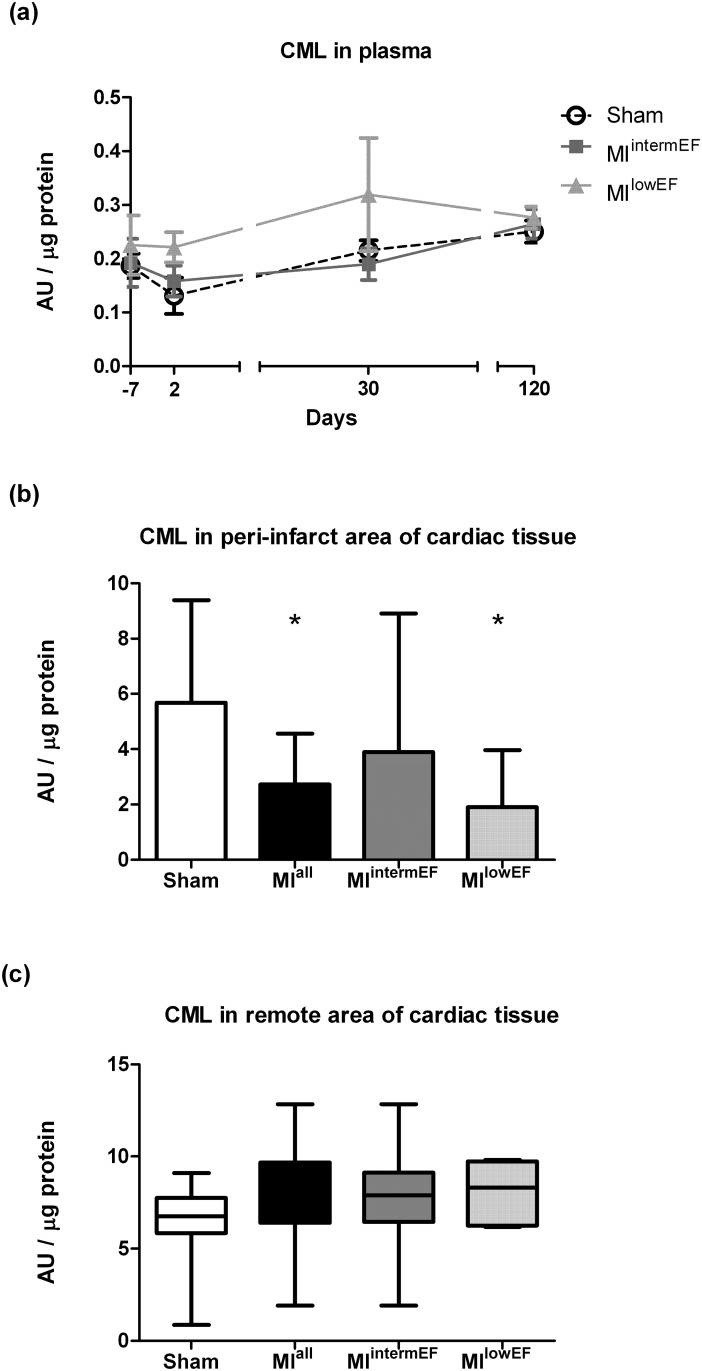
CML quantification by dot blot. Anti-CML antibody (2G11) was employed for AGE immunodetection in plasma (a) and cardiac tissue, both peri-infarct (b) and remote (c). Raw data available in the supporting material (S2 Fig). Statistical analysis: GEE (a), data expressed as geometric mean ± SE; ANOVA with Tukey’s test (b), data expressed as median ± IQR; Kruskal-Wallis with Dunn test (c). * Difference vs. sham (p < 0.01; sham group, n = 15; MI group, n = 7 per group). AU: arbitrary units. MI^intermEF^: EF > 53.7% and < 66.5%; MI^lowEF^: EF < 53.7.

No significant difference in CML concentrations was observed over time, whether in plasma (Fig 4a) or in remote heart tissue (Fig 4c). However, in the peri-infarct area, CML levels decreased in the MI group (Fig 4b). When MI rats were stratified by LVEF, only those in the MI^lowEF^ subgroup exhibited decreased levels when compared to the sham group.

### MI does not affect soluble RAGE levels in plasma, but increases RAGE expression in heart tissue

**Fig 5:**
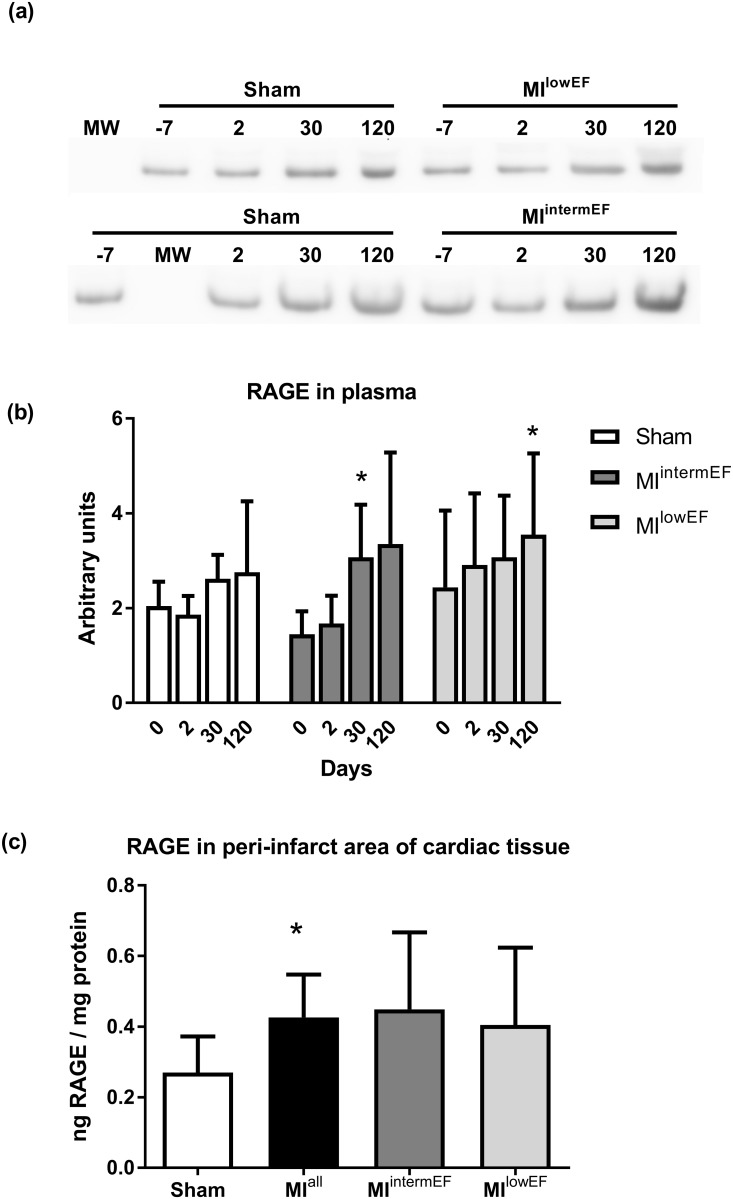
RAGE levels. sRAGE levels in plasma by Western blot (a, b) and RAGE levels in heart homogenate measured by ELISA (c). Representative image showing the signal at 45 kDa (a). Statistical analysis: GEE, data expressed as mean ± SE (* p < 0.01 compared with day zero in the same group); sham group, n = 5; MI^intermEF^ group, n = 5; MI^lowEF^ group, n = 6) (b). ANOVA with Tukey’s test, data expressed as geometric mean ± 95% CI (n = 15; MI group, n = 7 per group). * p = 0.052 compared with sham (c); MI^intermEF^: EF > 53.7% and < 66.5%; MI^lowEF^: EF < 53.7.

The soluble isoform of RAGE (sRAGE) was detected in plasma by Western blot, and no differences between groups were detected for each time point assessed. However, sRAGE levels increased in both MI groups over time (Fig 5b). Total RAGE levels in heart tissue increased approximately 50% in the MI group when compared with the sham group. However, no difference was observed when the analysis was performed considering LVEF (Fig 5c). This increase was not associated with the decrease in the levels of tissue CML (ρ = 0.28, p = 0.15) or yellowish-brown AGEs (ρ = 0.14, p = 0.47).

## Discussion

Increased levels of AGEs after MI may predict development of HF [11]. This suggests that anti-AGE drugs, such as pyridoxamine and aminoguanidine derivatives, might be employed as an adjuvant therapy in this setting [21,22]. However, before starting clinical studies, new therapies must undergo preclinical tests in reliable animal models. Using a consolidated animal model of MI, we demonstrated that neither circulating AGE nor RAGE levels increased during 12-week follow-up. Conversely, we found a reduction in AGEs and an increase in RAGE levels in heart tissue homogenate at the end of the follow-up period.

AGE formation depends on the chemical reaction between a free primary amine from a protein with a reactive carbonyl sugar. Free primary amines constitute a target site for oxidative modifications (e.g., carbonyl) and glycation reactions, according to the Amadori rearrangement mechanism [23]. Thus, decreased levels of reactive free amines and increased levels of protein carbonyl can represent AGE formation [24]. It has been demonstrated that post-infarct patients exhibit increased levels of plasma protein carbonyl shortly after hospital admission, which then decrease after 48 hours [25,26]. Using reactive free amine and carbonyl content as surrogate markers for protein damage, we found no significant changes between groups or over time. We also evaluated amine and carbonyl content in heart homogenates and demonstrated that, in this widely used animal model of MI, carbonyl levels did not increase and free reactive amine levels did not decline in heart tissue, regardless of LVEF or tissue region (peri-infarct or remote area).

Likewise, we were unable to demonstrate protein glycation in plasma by fluorescence emission. This parameter has already been found to be increased in post-MI patients and to predict HF development [11], despite not being increased after the disease was established [12]. Fluorescent AGE detection is widely employed in biological fluids [5, 11, 12, 17]. AGEs can also be detected by visualization of yellowish-brown compounds at 340 nm absorbance. In our sample, we found a signal decrease in plasma and tissue homogenates from MI rats with greater myocardial dysfunction (MI^lowEF^). Unfortunately, there are no data from clinical MI patients to compare with our findings.

CML is the best-characterized AGE and one of the most important RAGE ligands. Furthermore, it is the main product of *in vivo* oxidative cleavage of Amadori products and has been employed as a general marker of protein glycation [27]. In our sample, we did not find any increase in CML plasma levels from baseline during follow-up of MI rats. This is consistent with previous studies conducted in patients with ischemic heart disease [28]. Curiously, we found a decrease in CML content in heart tissue from MI rats. Some studies employing immunohistochemistry and Western blot analysis have demonstrated an increase in AGE levels in cardiac tissue from rats subjected to experimental MI induction [29–31]. AGEs are heterogeneous and differences in the targets chosen to analyze may partly explain our disagreement with previous works in animal model of MI. Besides, it is important to note that Amadori products might artificially convert into AGEs by high-temperature treatment of histological sections, leading to false labeling of the samples [32], a bias that is not present in our study.

RAGE plays a role in several cardiovascular diseases, as a trigger of inflammation and oxidative pathways. Its soluble form, sRAGE, may act both as circulating biomarker and as AGE-quencher, impairing AGE signaling in cellular RAGE [33,34]. It has been shown that sRAGE plasma level is a potential marker of HF severity in ischemic patients [35]. In one study, HF patients exhibited a 14% increase in sRAGE plasma levels compared to controls [36]. We were unable to identify sRAGE in plasma samples, but RAGE levels in heart homogenate were approximately 50% higher 120 days after MI. Statistical significance was lost when RAGE levels were assessed in LVEF-stratified subgroups, however our small sample size could explain this phenomenon. Cao et al. (2014) found increased RAGE production in the heart 4 to 12 weeks after LAD ligation in rats [30]. This study employed Western blot to assess RAGE levels, which is a semi-quantitative approach. Thus, comparisons with our study must be performed with caution.

Few studies, whether in humans or animals, have investigated AGEs and RAGE after MI, and none was designed to describe AGE and RAGE levels in a time-course manner. The present study provides the first descriptive analysis of AGE formation and RAGE expression after MI and comparisons can be made with either previous human or animals studies.

When compared with MI or HF patient studies, we notice the animal model fail in representing what happens in real life. Studies usually employ AGE/RAGE levels as biomarkers for future outcomes and do not compare with a healthy control [10–12, 35–37]. We performed a case-control study, with a 4-month follow up and could not find any association between early AGE/RAGE levels and the worsening ejection fraction, fractional shortening, or chamber volume.

When compared with other animal model studies, we see some disagreements: whereas others saw increased AGEs, we saw unchanged circulating levels and even decreased heart levels of AGEs during a long follow up. However, it is important to notice that AGEs are heterogeneous and antibodies raised against AGEs may not detect some specific molecules. That is a main limitation when comparing other studies with ours.

Some technical limitations of the present study must be addressed. First, high-performance liquid chromatography–mass spectrometry (LC/MS) is considered the most accurate approach to detect AGEs, such as CML, CEL, and pentosidine. However, access to such equipment is still limited to few laboratories. Despite this limitation, we used robust methods in our analysis (a commercially available ELISA kit and purified anti-CML and anti-CEL antibodies). We recognize that other glycation intermediates and stable modifications could have been assessed to better characterize this animal model; however, we chose to focus on the most important ones. Second, harvesting cardiac tissue over time would have allowed a more complete characterization of AGE formation and RAGE expression in the myocardium during cardiac remodeling. Although RAGE levels in tissue homogenate had not increased by the end of follow-up in our sample, it would have been interesting to assess whether variation occurred throughout the study, mainly in the first days after MI.

In this study, we characterized the formation of AGEs and the expression of RAGE in the plasma and myocardial tissue of post-MI Wistar rats free of comorbidities and environmental or behavioral factors. We found a decrease in yellowish-brown AGEs and CML after MI in myocardial tissue and an increase in tissue RAGE levels. However, no increase in plasma AGEs or sRAGE was observed, which suggest that the rat MI model should be employed with caution when studying the AGE-RAGE signaling axis or anti-AGE drugs.

## Supporting information

S1 Fig Animal distribution and losses during the study. Deaths recorded at any time point between the anesthesia and animal recovery after the surgery (5 hours after the anesthesia) were categorized as ‘Surgical deaths’. Exclusion criteria were: i) MI animal with < 30% of akinesia/hypokinesia detected in the first ultrasound analysis (48 hours after MI surgery) and ii) sham animal with signs of akinesia/hypokinesia detected in any ultrasound analysis during the follow-up.
(TIF)

S2 Fig Dot blot images. (DOCX)

S3 Fig RAGE immunocontent in remote and peri-infarct regions of myocardium. Sample (20 μg) was loaded in SDS-PAGE gel (10%) and run at 120 V. After electrotranferring to a PVDF membrane, Coomassie staining was performed and registered to use as loading control. This analysis was performed in the available biological sample after the reviewer request and the sample size is the following: Remote region: 6, 12, 5, and 7 for Sham, MI^all^, MI^intermedEF^, and MI^lowEF^, respectively. Peri-infarct region: 4, 9, 4, and 5 for Sham, MI^all^, MI^intermedEF^, and MI^lowEF^, respectively. As Sham animals do not have peri-infarction region, we collected myocardium from the LV to use as comparative. Remote region stands for myocardium from septum. Data is shown as IQR and median. p > 0.05 (Kruskal-Wallis). MI^intermEF^: EF > 53.7% and < 66.5%; MI^lowEF^: EF < 53.7.
(TIF)
