# Supplementary figures and images for: Characterization of advanced glycation end products and their receptor (RAGE) in an animal model of myocardial infarction
Source: PLoS One. 2019 Jan 11;14(1):e0209964. doi: 10.1371/journal.pone.0209964 (PMC6329515; doi:10.1371/journal.pone.0209964)

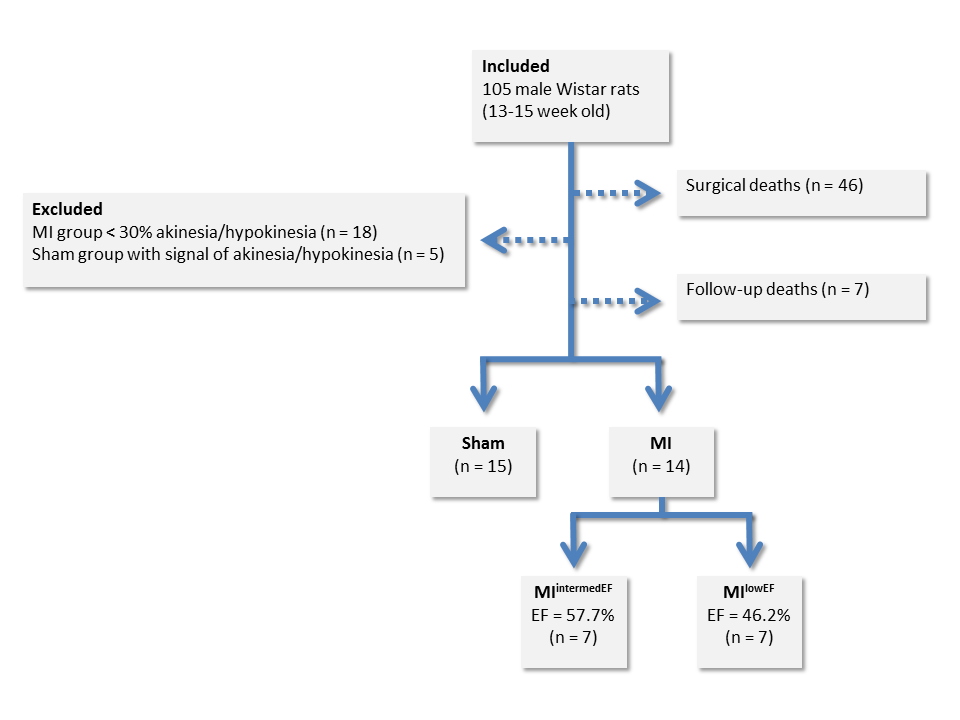

Supplement: S1 Fig — Deaths recorded at any time point between the anesthesia and animal recovery after the surgery (5 hours after the anesthesia) were categorized as ‘Surgical deaths’. Exclusion criteria were: i) MI animal with < 30% of akinesia/hypokinesia detected in the first ultrasound analysis (48 hours after MI surgery) and ii) sham animal with signs of akinesia/hypokinesia detected in any ultrasound analysis during the follow-up. (TIF) [file pone.0209964.s001.tif]

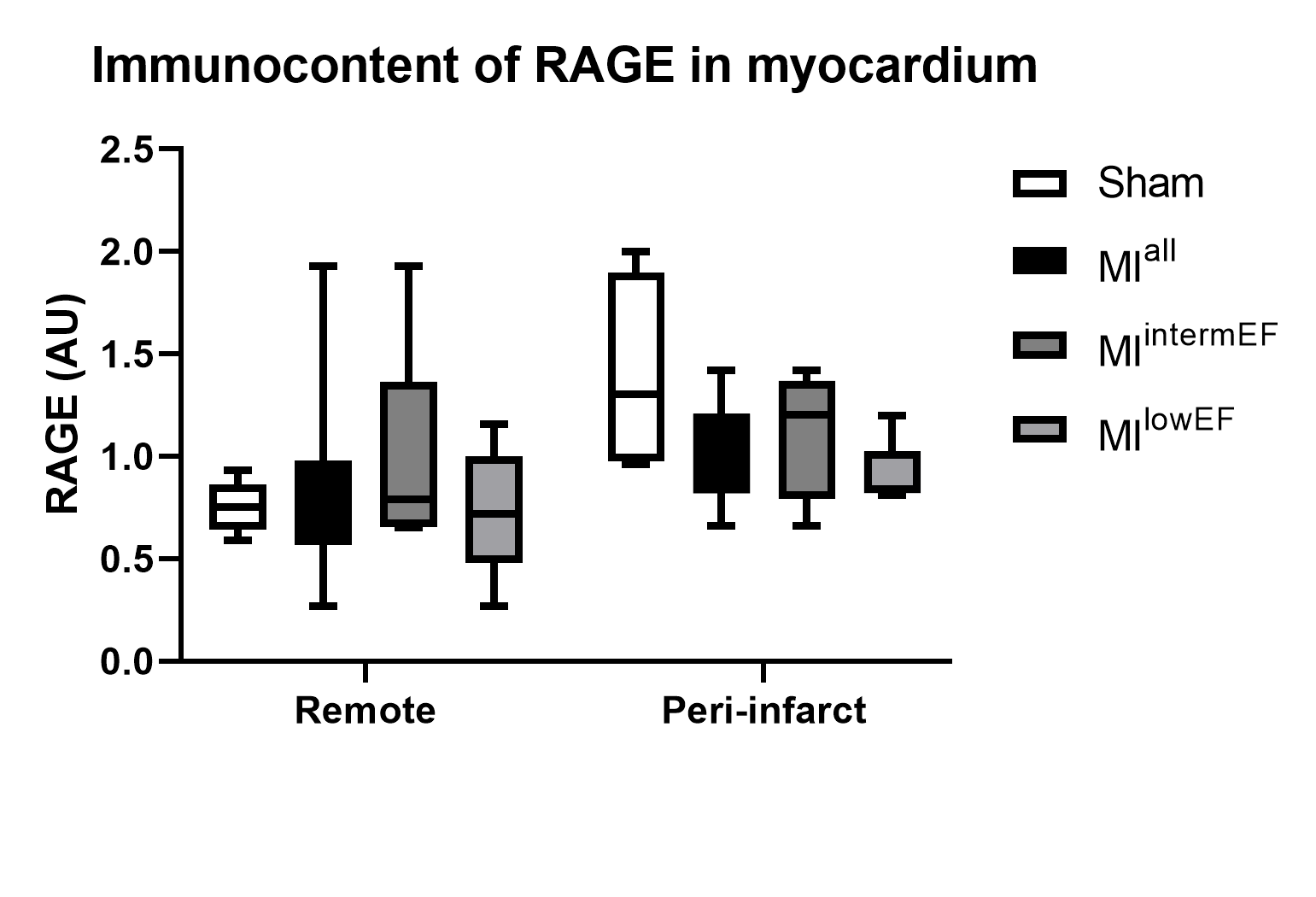

Supplement: S3 Fig — Sample (20 μg) was loaded in SDS-PAGE gel (10%) and run at 120 V. After electrotranferring to a PVDF membrane, Coomassie staining was performed and registered to use as loading control. This analysis was performed in the available biological sample after the reviewer request and the sample size is the following: Remote region: 6, 12, 5, and 7 for Sham, MIall, MIintermedEF, and MIlowEF, respectively. Peri-infarct region: 4, 9, 4, and 5 for Sham, MIall, MIintermedEF, and MIlowEF, respectively. As Sham animals do not have peri-infarction region, we collected myocardium from the LV to use as comparative. Remote region stands for myocardium from septum. Data is shown as IQR and median. p > 0.05 (Kruskal-Wallis). MIintermEF: EF > 53.7% and < 66.5%; MIlowEF: EF < 53.7. (TIF) [file pone.0209964.s003.tif]
