# Supplementary material for: Characterization of advanced glycation end products and their receptor (RAGE) in an animal model of myocardial infarction
Source: PLoS One. 2019 Jan 11;14(1):e0209964. doi: 10.1371/journal.pone.0209964 (PMC6329515; doi:10.1371/journal.pone.0209964)

Heart homogenates (a) or plasma samples (b) were were blotted in PVDF membranes and probed with 2G11 antibody . Samples disposition is shown in (c). N stands for a normalization sample.

a)
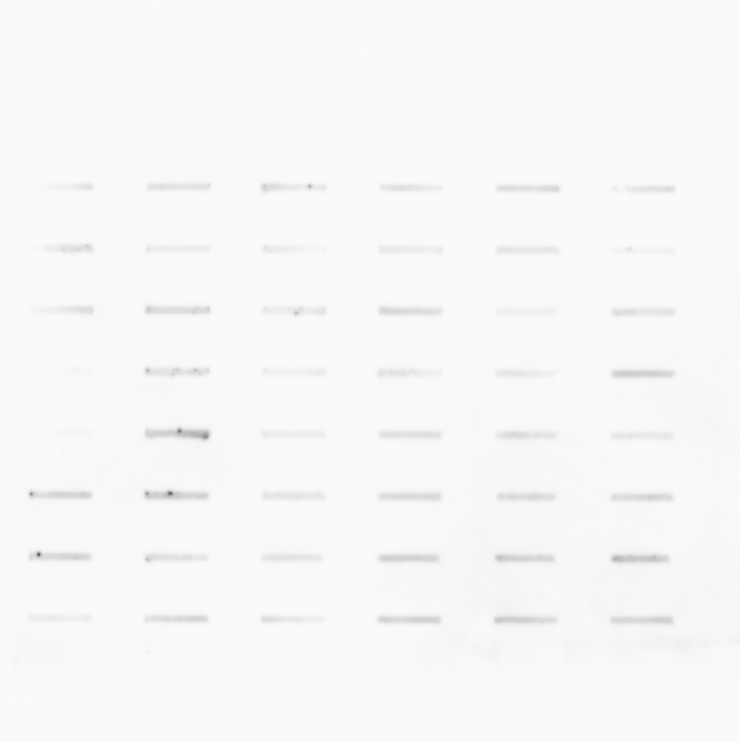


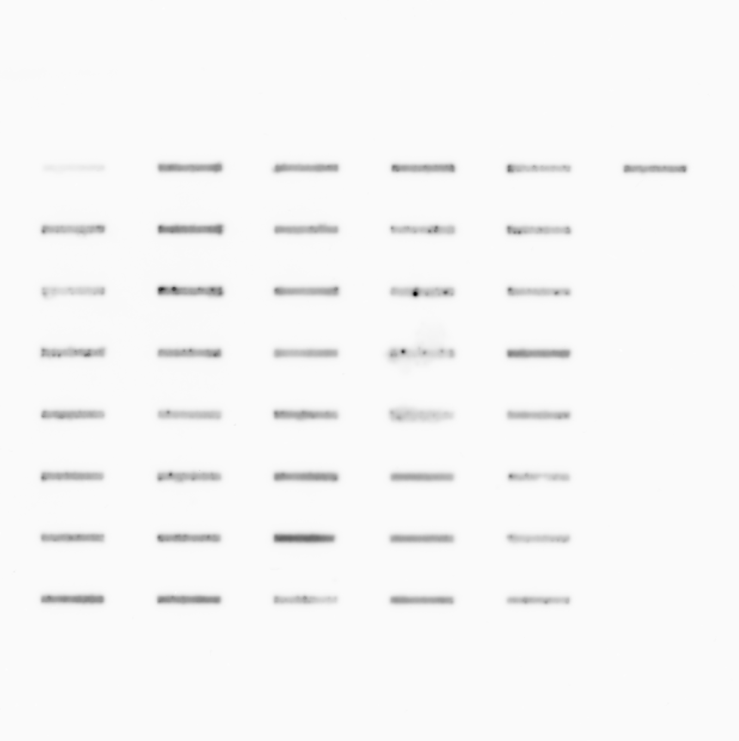


b)


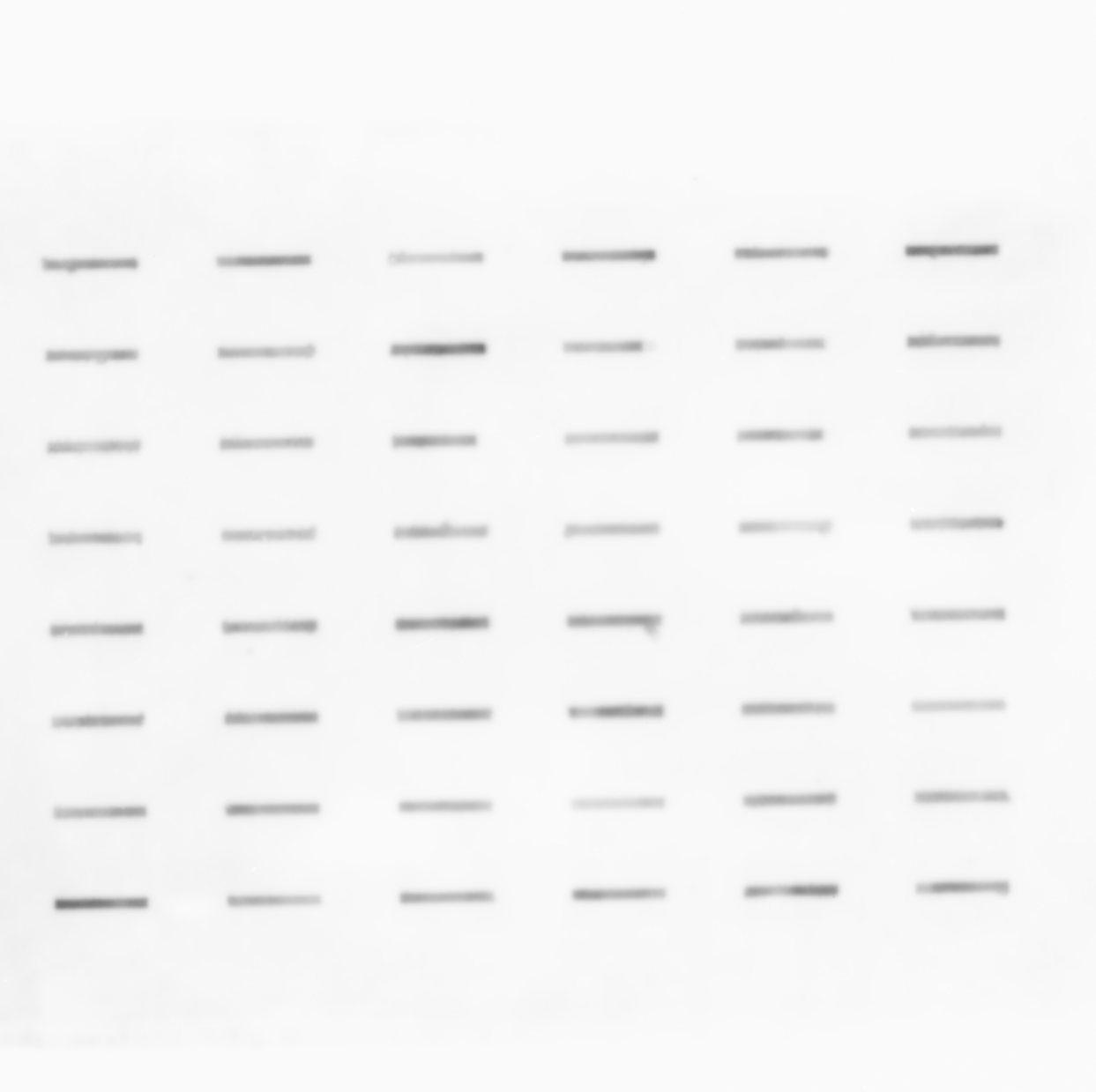

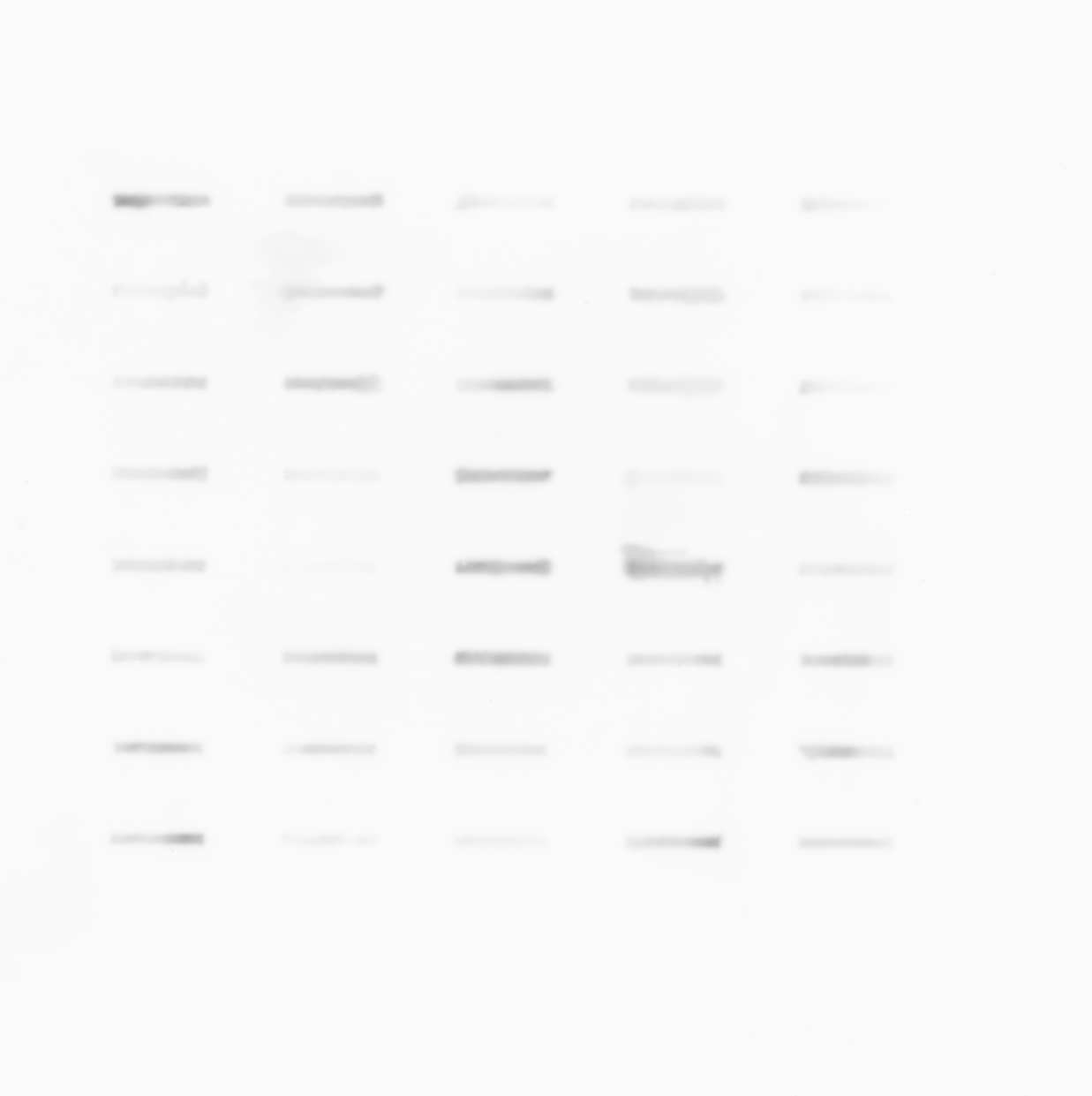


c)


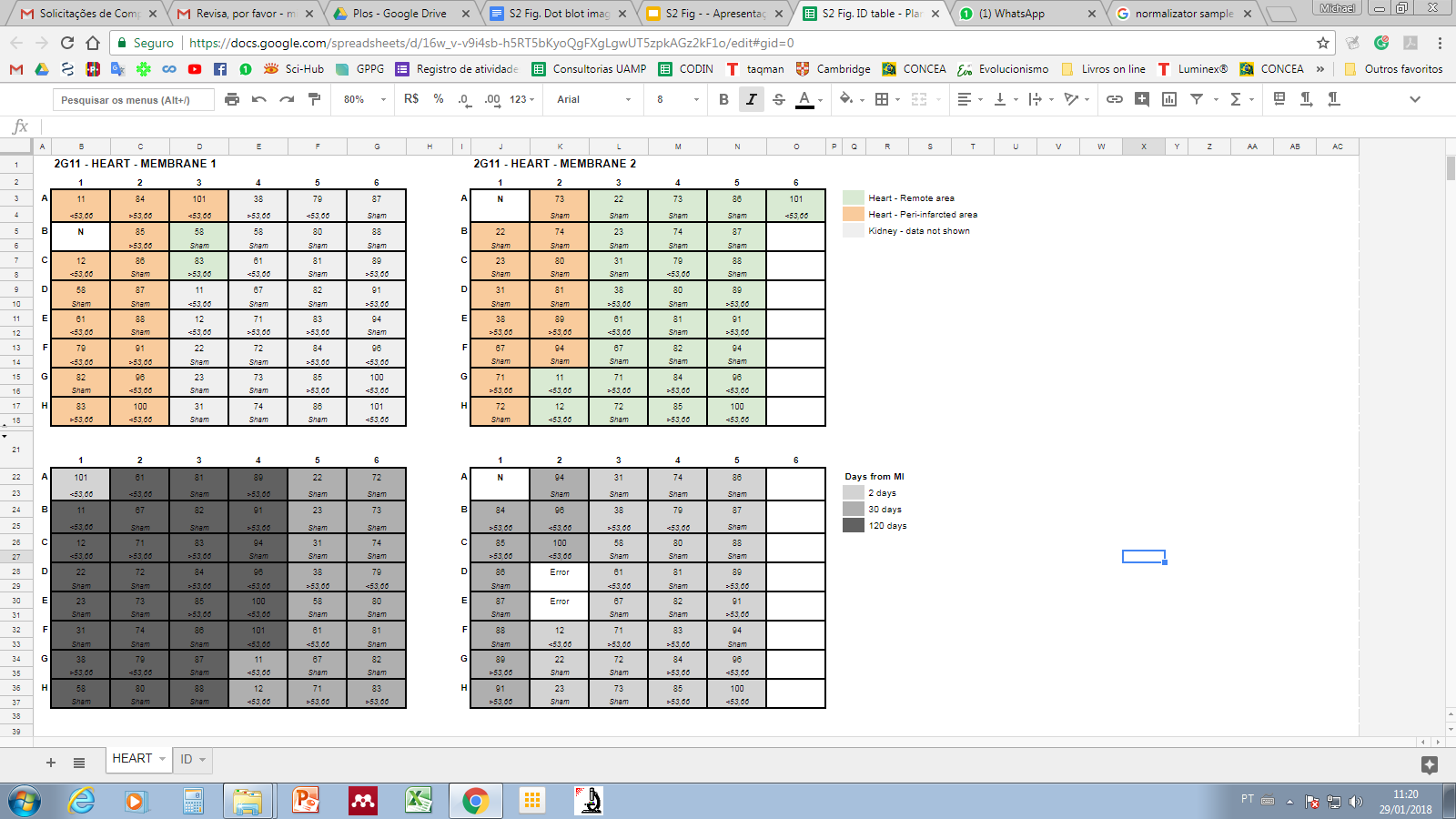

Supplement: S2 Fig — (DOCX) [file pone.0209964.s002.docx]
